# Transcriptome profiling revealed diverse gene expression patterns in poplar (*Populus* × *euramericana*) under different planting densities

**DOI:** 10.1371/journal.pone.0217066

**Published:** 2019-05-29

**Authors:** Kun Ning, Changjun Ding, Qinjun Huang, Weixi Zhang, Chengchao Yang, Dejun Liang, Ruting Fan, Xiaohua Su

**Affiliations:** 1 State Key Laboratory of Tree Genetics and Breeding, Research Institute of Forestry, Chinese Academy of Forestry; Key Laboratory of Tree Breeding and Cultivation, State Forestry and Grassland Administration, Beijing, China; 2 Liaoning Provincial Institute of Poplar, Gaizhou, Liaoning Province, China; 3 Annoroad Gene Technology Co., Ltd, Beijing, China; 4 Co-Innovation Center for Sustainable Forestry in Southern China, Nanjing Forestry University, Nanjing, Jiangsu Province, China; ICAR-Indian Institute of Agricultural Biotechnology, INDIA

## Abstract

Certain plant genotypes can achieve optimal growth under appropriate environmental conditions. Under high planting density conditions, plants undergo competition for uptake and utilization of light and nutrients. However, the relationship between whole-genome expression patterns and the planting density in perennial woody plants remains unknown. In this study, whole-genome RNA sequencing of poplar (*Populus* × *euramericana*) was carried out at three different sampling heights to determine gene expression patterns under high (HD) and low (LD) planting densities. As a result, 4,004 differentially expressed genes (DEGs) were detected between HD and LD, of which 2,300, 701, and 1,003 were detected at the three positions, upper, middle and bottom, respectively. Function annotation results further revealed that a large number of the DEGs were involved in distinct biological functions. There were significant changes in the expression of metabolism-related and stimulus-related genes in response to planting density. There were 37 DEGs that were found at all three positions and were subsequently screened. Several DEGs related to plant light responses and photosynthesis were observed at different positions. Meanwhile, numbers of genes related to auxin/indole-3-acetic acid, and carbon and nitrogen metabolism were also revealed, displaying overall trends of upregulation under HD. These findings provide a basis for identifying candidate genes related to planting density and could increase our molecular understanding of the effect of planting density on gene expression.

## Introduction

Tree growth is closely related to environmental conditions. For example, planting density affects population structure and yield. An optimal planting density allows effective utilization of light energy and soil nutrients, ensuring normal development of individual plants and coordinated development of the entire population. In line with this, planting density has been shown to improve grain yield [1,2], via its effect on plant size, morphology, biomass, productivity, and the extent of lodging [3,4]. The density of planting has also been found to be important in acquiring a high yield in modern corn production [5,6]. Although planting density has been shown to be important for the growth of crops, vegetables, and medicinal plants [7], little is known about the molecular-level effect of planting density on plant growth.

Planting density stress elicits a competitive response for light between neighboring plants [8]. Light, the driving force of photosynthesis, also affects photosynthetic function by regulating leaf development and morphological structure [9]. Light is a key environmental factor and an important consideration for agroforestry production [10,11]. Under high planting density, plants increase light capture by expanding their canopy to elevate yield [12]. Studies have also shown that with increasing density, light transmittance to each layer decreases significantly, ultimately affecting yield [13]. Nevertheless, the regulatory mechanism involved in the gene network response to light in woody trees under different planting densities remains largely unknown.

Poplar is the preferred species used in bioenergy plantations in temperate climates [14], mainly due to its high growth rate, timber yield, coppicing ability and adaptability to differing environmental conditions [15–17]. Studies on the response of trees to initial spacing (planting density) have previously focused on growth traits such as height, diameter at breast height (DBH), canopy structure, stem volume, and above-ground biomass production [18,19]. However, to fully maximize productivity, it is important to understand the molecular mechanisms and environmental factors that affect timber yield. RNA-sequencing (RNA-Seq) of *Arabidopsis thaliana* grown under high and low planting densities revealed that the expression of glutaredoxin genes was influenced by the planting density the most [7]. Moreover, several genes associated with yield heterosis were found to exhibit differences in transcript accumulation in maize grown under high and low planting densities [20]. We therefore utilized RNA-Seq to detect whole-genome expression patterns in poplar under different planting densities with the aim of identifying density-regulated genes.

## Materials and methods

### Plant materials and sampling collection

Nine-year-old plants of *Populus* × *euramericana* ‘BF3’ were used as experimental materials. Selection of the ‘BF3’ line was based on its optimal performance as well as its certification as a new improved forest variety. The DBH growth of nine-year-old poplar trees was measured at the end of growing season with a tree breast diameter ruler. A randomized block design was used to establish the trial with three replicates. Two planting densities were set up in the trial, high (HD) and low (LD). The HD block contained 18 trees (6 × 3 rows) in each 2 × 5 m plot. The LD block contained 15 trees (5 × 3 rows) in each 4 × 8 m plot. Sampling height was divided into three equally-spaced vertical positions (bottom-B, middle-M, and upper-U). Leaf tissues were sampled (three positions per tree, one tree per block) from four directions (two leaves in each direction, east, south, west and north, respectively) in the innermost tree of the block during the most rapid growth stage. The samples were frozen in dry ice and then stored at -80°C in a freezer.

### RNA extraction, cDNA library construction and RNA sequencing

Total RNA was extracted using TRIzol Reagent (Invitrogen, USA) according to the manufacturer’s protocol. RNA integrity and concentration were determined using an Agilent 2100 RNA Nano 6000 Assay Kit (Agilent Technologies, CA, USA). Sequencing libraries were generated using a NEBNext Ultra RNA Library Prep Kit for Illumina (#E7530L, NEB, USA) according to the manufacturer’s instructions. The cDNA libraries were pooled and sequenced using an Illumina NovaSeq 6000 platform (Illumina, USA) at Annoroad Gene Technology Co., Ltd (Beijing, China).

### Bioinformatic analysis

Fastq files were acquired using an NGS QC Toolkit [21]. Filtered high-quality clean reads were aligned to the reference genome of *Populus deltoides* Marsh (JGI 2.1) using HISAT2 software [22]. Analysis of gene expression levels was carried out by HTSeq (0.6.0) using the fragments per kilobase of transcript per million mapped fragments (FPKM) method [23]. Differentially expressed genes (DEGs) were screened based on a threshold of an absolute fold change of ≥1.5 and a *P* value of ≤0.05 [24]. Clean reads were compared against the National Center for Biotechnology Information (NCBI) non-redundant (NR) protein sequence database and nucleotide (NT) sequence database using Blast X. The reads were aligned using the public universal protein (UniProt) and protein family (Pfam) databases. Gene Ontology (GO) enrichment analysis of DEGs was performed using the GOseq R package [25]. Corrected *P*-values less than 0.05 were considered as significantly enriched GO terms. KOBAS software was implemented to test the statistical enrichment of DEGs in the Kyoto Encyclopedia of Genes and Genomes (KEGG) pathways [26].

### Quantitative real-time PCR analysis

Quantitative real-time (qRT)-PCR analysis was performed to verify the accuracy of the transcriptome profiles. Analysis of gene expression levels of 10 randomly selected genes was carried out. qRT-PCR was performed using a LightCycler 480 Instrument II system (Roche, Switzerland) and analyzed with SYBR Premix Ex Taq II (Takara) using the following parameters: 95°C for 30s, 40 cycles of 95°C for 5s and 60°C for 30s, followed by 95°C for 5s, 60°C for 1 min, and 95°C with continuous acquisition mode at 5 per°C, with a final extension at 50°C for 30s. Three replicates were performed for each sample. Relative expression levels were calculated using the 2^-ΔΔCt^ method [27]. *β-actin* was used as an internal reference (specific primers 5’-CATCCAGGCTGTCCTTTCCC-3’; 5’-AACGAAGGATGGCGTGTGG-3’). The PCR primers for genes tested from the RNA-Seq data are listed in S1 Table.

## Results

### Measurement of plant DBH growth

**Fig 1:**
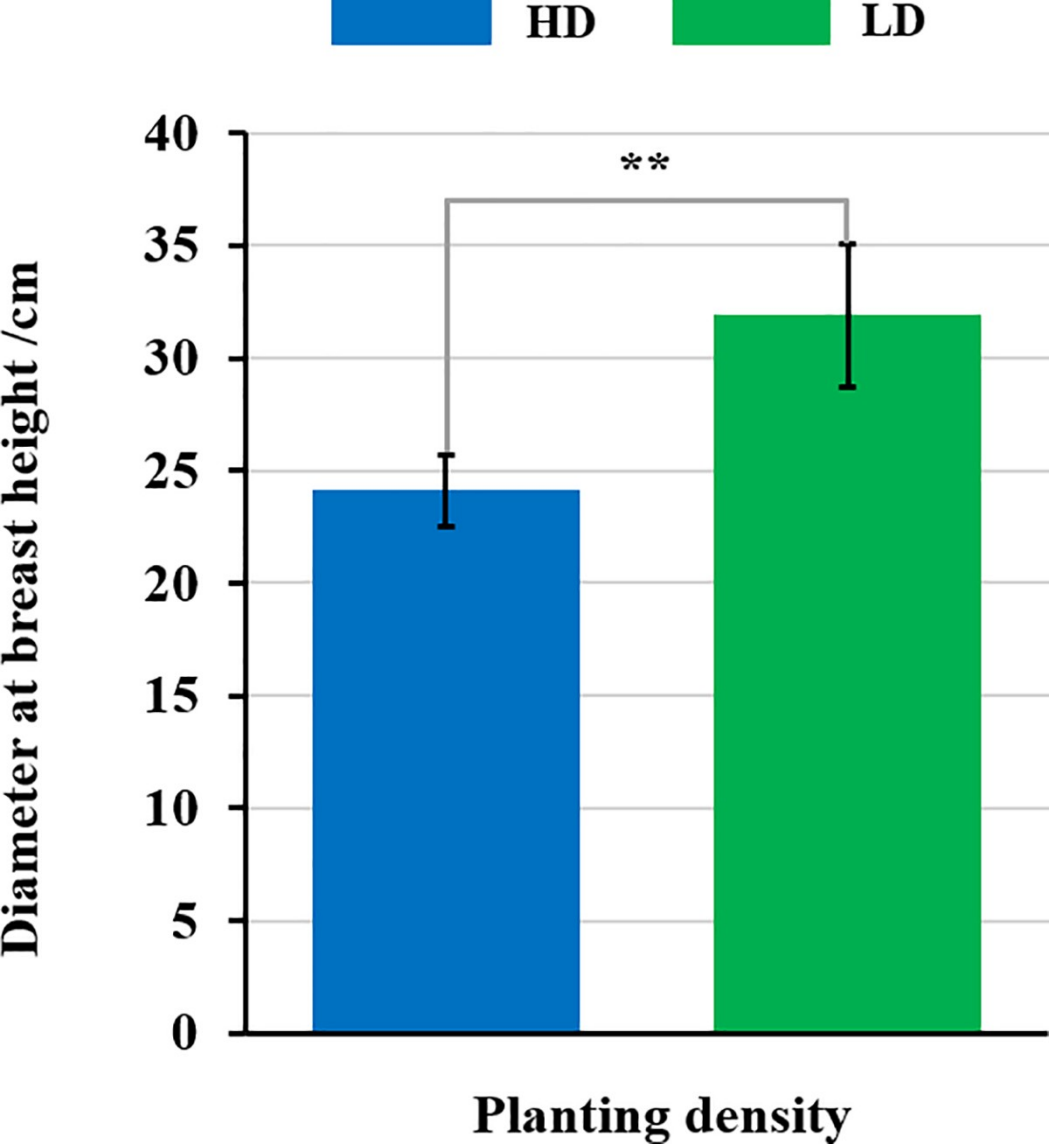
Analysis of Diameter at Breast Height (DBH) growth of poplar under different planting densities. Nine-year-old poplar trees were grown under high (HD) and low (LD) planting density and their DBH was measured.** indicates significant difference at a level of 0.01 by one-way ANOVA using a Duncan *t* test.

The DBH growth of nine-year-old poplar trees grown under two planting densities was highly significantly different (*p* <0.01); the DBH of trees grown under LD was significantly higher than those grown under HD (Fig 1). For poplar, trees grown under HD are already in a canopy closure state at the age of nine, while under LD, trees have not yet reached canopy closure. Canopy closure can dramatically changes the growth conditions and finally affects the trees’ growth and development.

### Overview of RNA sequencing

To identify the gene expression patterns in poplar under HD and LD, 18 leaf samples were obtained from two planting densities at three positions and subjected to RNA-seq analysis. The average number of clean reads generated from triplicate libraries was 45,157,000 (~13.5 Gb) and 44,532,724 (~13.4 Gb) under HD and LD, respectively. The overall average clean read rate was 96%, with an approximate quality score of 93% clean Q30 bases (S2 Table). A total of 87% of the clean reads could be mapped to the reference genome of *P*. *deltoids*, with the average ratio of uniquely mapped reads exceeding 81%, and the multi-mapped rate at less than 6% (S3 Table). The distribution region of the mapped reads showed that most reads were mapped in the exon region (~85%), followed by intergenic (~10%) and intron regions (~5%) (S4 Table).

### Analysis of the expressed genes

For quantification of gene expression levels in the RNA-Seq data, the FPKM method was used to account for the effect of sequencing depth and gene length on the fragment counts. The gene numbers at three different expression values (FPKM levels) were counted (S5 Table). The results revealed that approximately 90% of genes had a FPKM level >0, while ~23% had a low expression value of 0 <FPKM ≤1. Furthermore, ~64% had a moderate expression value of 1 <FPKM ≤100, while only ~3% had a high expression value of FPKM >100.

To determine the reliability and rationality of sample selection, the correlation coefficient of gene expression levels between samples was calculated based on the FPKM values. As a result, significant high correlations were revealed between each pair of samples, all of which were greater than 0.95, indicating relatively good repeatability (S1 Fig).

**Fig 2:**
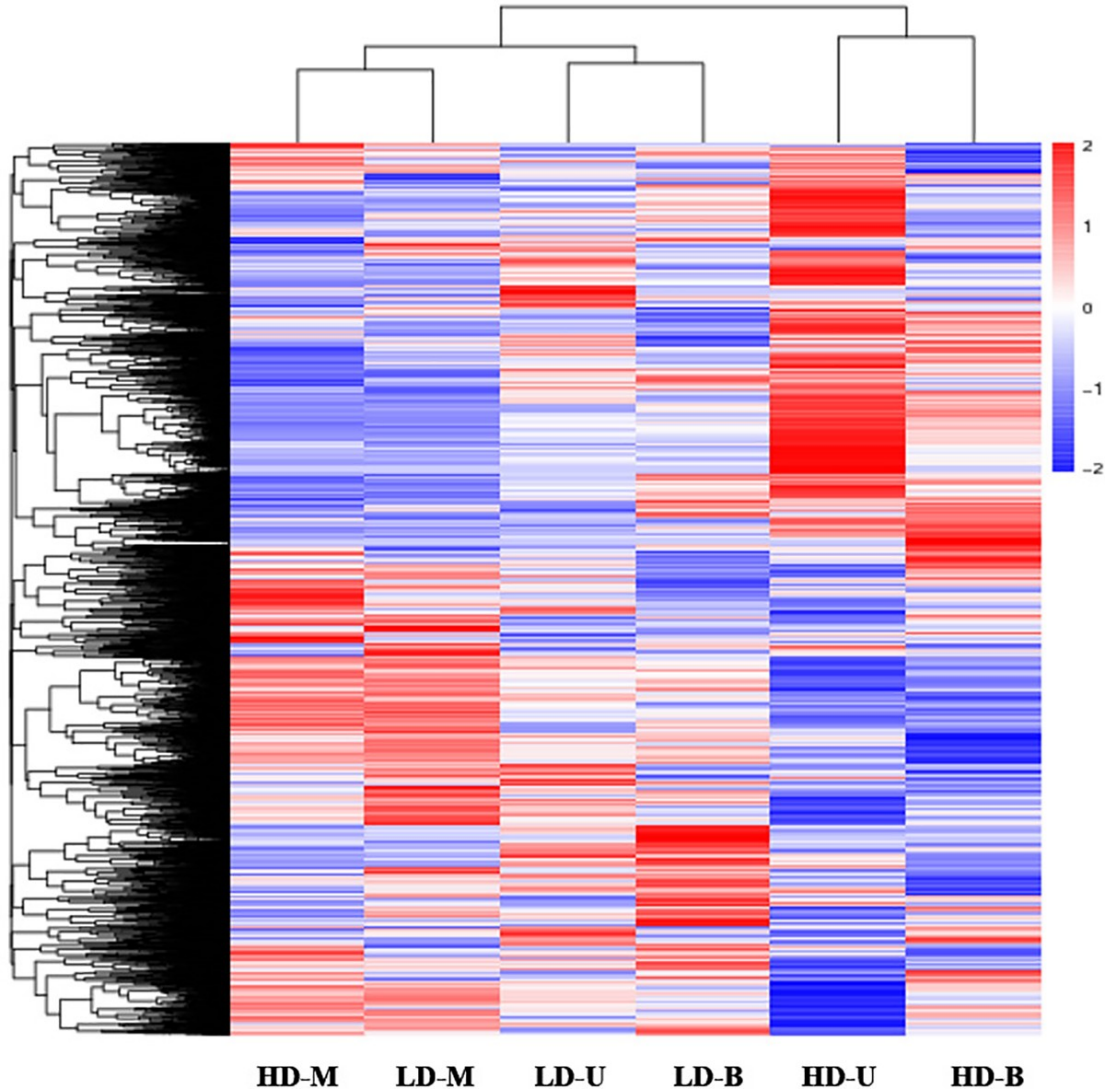
Heatmap showing gene expression at three vertical positions on poplar trees under different planting densities. Gene expression was analyzed at three positions on poplar trees, upper (U), middle (M) and bottom (B), that were grown under two planting densities, high (HD) and low (LD). Blue indicates low expression levels and red indicates high expression levels.

To further understand the expression profiles of the identified genes, a heatmap was obtained (Fig 2). The clustering results showed that genes from the middle position in HD and LD clustered into a single branch, while genes from the upper and bottom positions in each planting density gathered together.

### Quantification of DEGs under different planting densities

**Fig 3:**
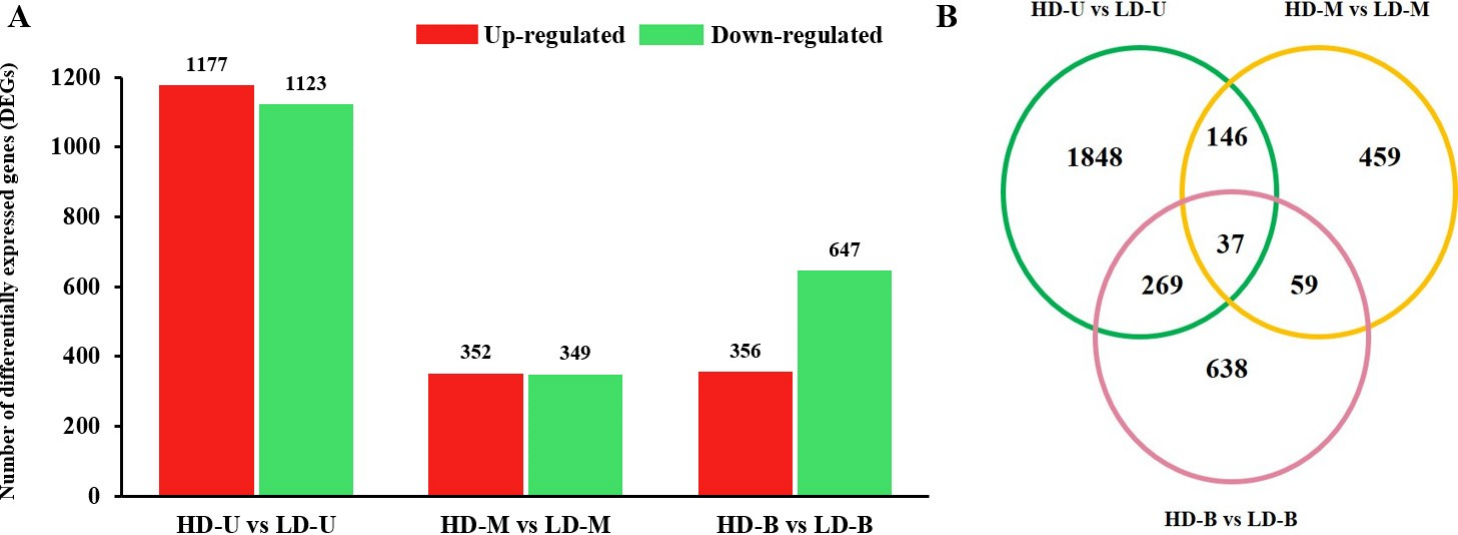
Gene expression profiles of differentially expressed genes (DEGs) in all comparison groups. Samples were taken from poplar trees at three vertical positions: upper (U), middle (M) and bottom (B). The trees were grown under high (HD) and low planting density (LD). (A) The number of DEGs between HD and LD for three different comparisons (one for each vertical position). The red bars represent the number of upregulated DEGs under HD, while the green bars represent the number of DEGs downregulated under HD. The vertical ordinate represents the number of DEGs. (B) Distribution of the DEGs identified when HD is compared to LD that are common and specific to the upper, middle, and bottom positions.

Of all the expressed genes, 4,004 DEGs were detected by means of three different comparisons (Fig 3A). Of these, 1,885 were upregulated and 2,119 were downregulated under HD. There were more DEGs between HD and LD in the upper position, than in the middle and bottom positions. To identify the DEGs between HD and LD at the three different positions, we analyzed the results of the three comparisons to obtain the DEGs specific or common to the transcriptome at each position. There were 37 DEGs common to all three comparisons (Fig 3B). Furthermore, the Venn diagram showed that 1,848 genes were specific to the comparison between HD and LD in the upper position, while a total of 459 and 638 unique DEGs were detected at the middle and bottom positions, respectively.

### Function annotation of the DEGs

**Fig 4:**
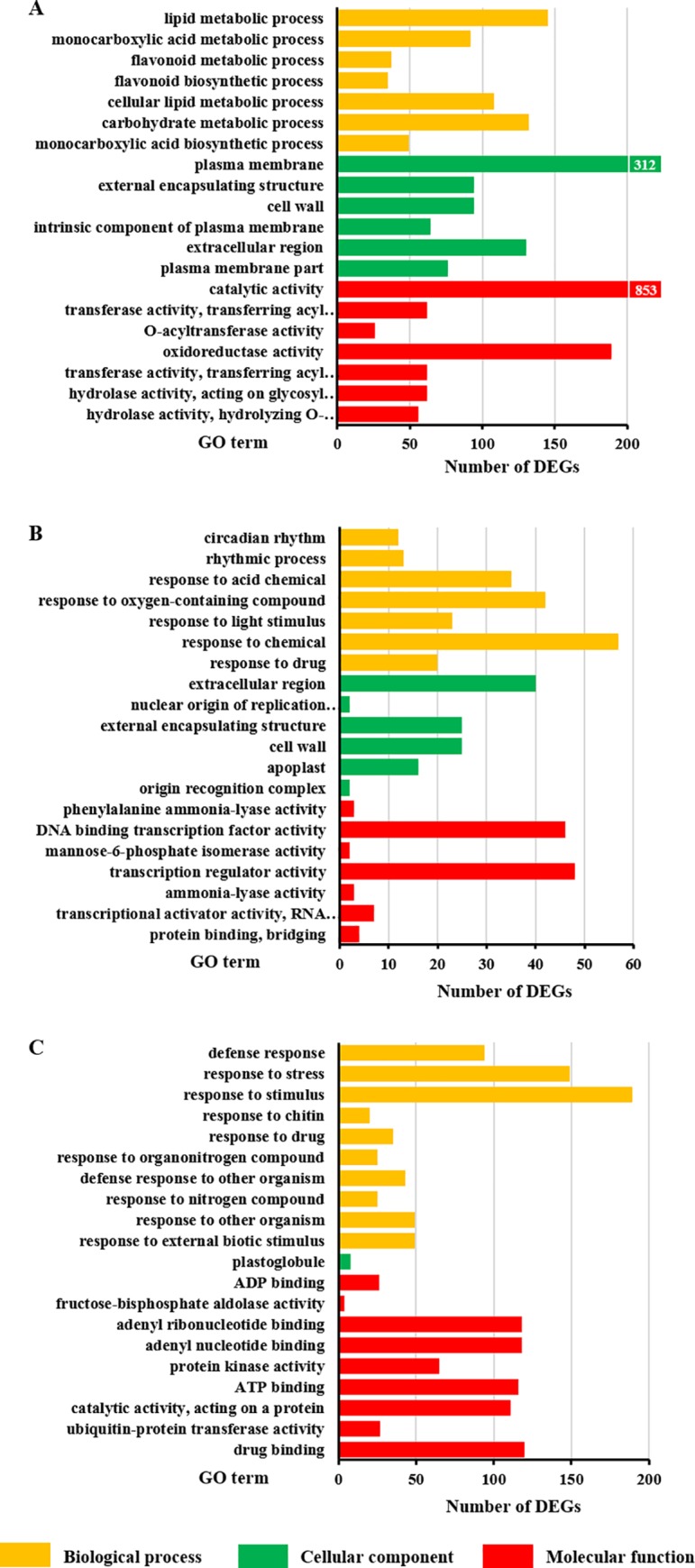
Gene Ontology (GO) enrichment analysis of differentially expressed genes (DEGs) unique to each vertical position. DEGs were identified by comparing samples from poplar trees planted under high (HD) and low (LD) density. The samples were taken from three vertical positions for the comparisons: upper (U), middle (M), and bottom (B). The top 20 most enriched GO terms of the DEGs unique to each vertical position are shown: upper (A), middle (B) and bottom (C). Yellow bars represent “biological process,” green represent “cellular component,” and red represent “molecular function” GO categories. All terms had a false discovery rate (FDR) <0.05, except for “cellular component” terms in the middle and bottom density comparisons (FDR <0.1).

The DEGs unique to each position were annotated using GO analysis and categorized into multiple GO terms (Fig 4). These DEGs belonged to three main categories: “biological process”, “cellular component”, and “molecular function.” The significantly enriched GO terms that appeared at the three vertical positions showed a divergent functional classification. We found that DEGs classed in the “biological process” category from the upper position, were mainly termed as “metabolic” and “biosynthetic” processes (Fig 4A), while at the middle and bottom positions, the DEGs were mainly involved in the response to different substances–the “response to” subcategories (Fig 4B and 4C). Additionally, there was visible diversity in the “cellular component” and “molecular function” categories. When comparing the two planting densities, 23 DEGs from the middle position were found in the “response to light stimulus” subcategory (Fig 4B). These results indicate that the DEGs found when comparing HD and LD at different positions, were involved in distinct biological functions.

**Fig 5:**
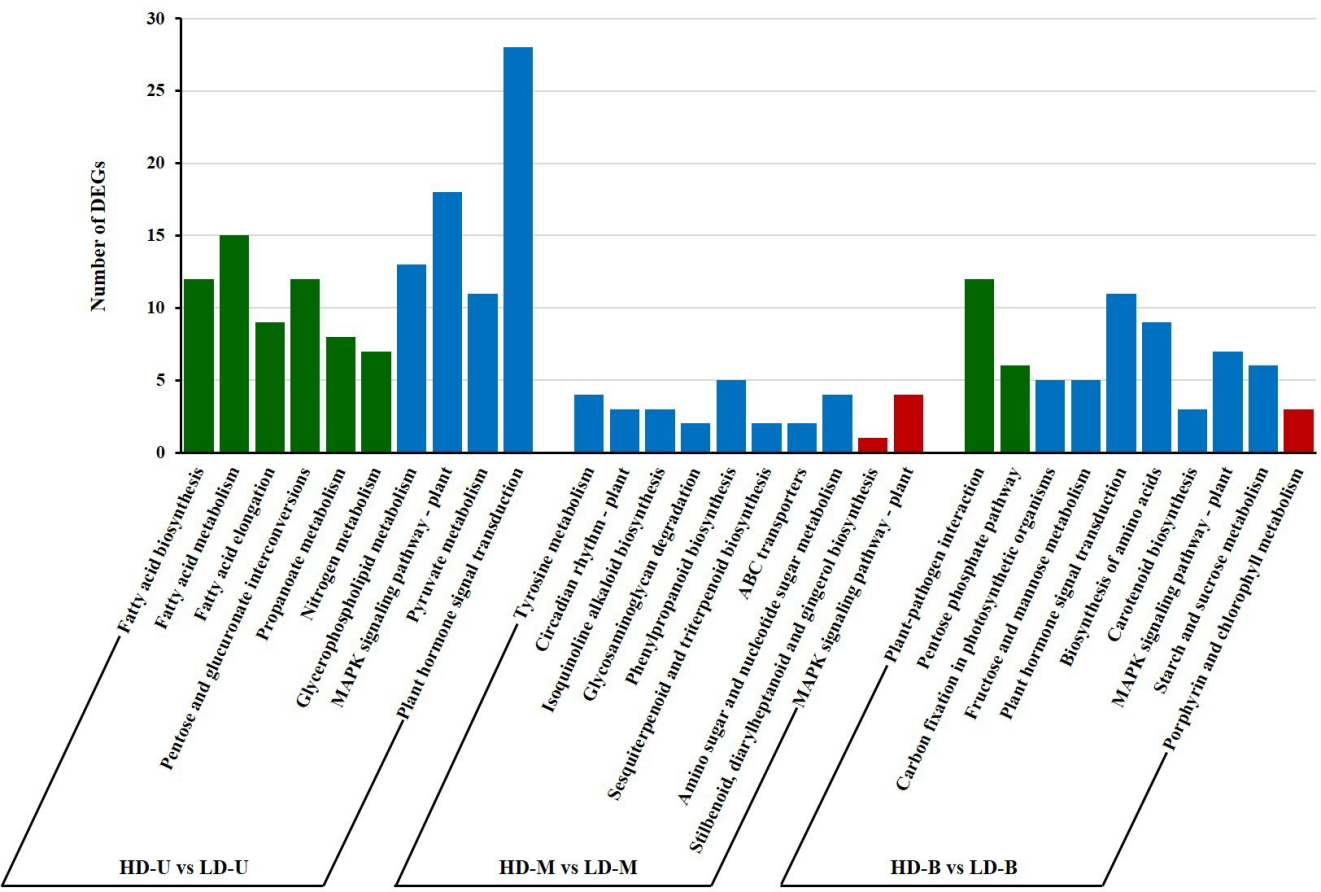
The top 10 KEGG enrichments for differentially expressed genes (DEGs) found when comparing planting densities. DEGs were identified by comparing samples from poplar trees planted under high (HD) and low (LD) density. The samples were taken from three vertical positions for the comparisons: upper (U), middle (M), and bottom (B). The top 10 most enriched KEGG pathways of the DEGs unique to each vertical position are shown. The bars colored green, blue, and red indicate the KEGG pathways with different enrichment levels (*q* <0.05, *p* <0.05 but *q* >0.05, *p* <0.1, respectively).

To investigate the pathways of the DEGs unique to each position, the enrichment of KEGG pathways was analyzed. The top 10 enriched pathways when comparing HD and LD are shown in Fig 5. For DEGs found at the upper position, fatty acid “biosynthesis,” “metabolism,” and “elongation” were the top three pathways with lower *q* value, however, “plant hormone signal transduction” had the most DEGs (28). In the middle group, each pathway was enriched with a relatively small number of DEGs. “Plant-pathogen interaction”, “pentose phosphate pathway” and “carbon fixation in photosynthetic organisms” were the three major enriched pathways with the lower *q* value for DEGs from the bottom position comparison.

### DEGs found at all three vertical sample positions

**Fig 6:**
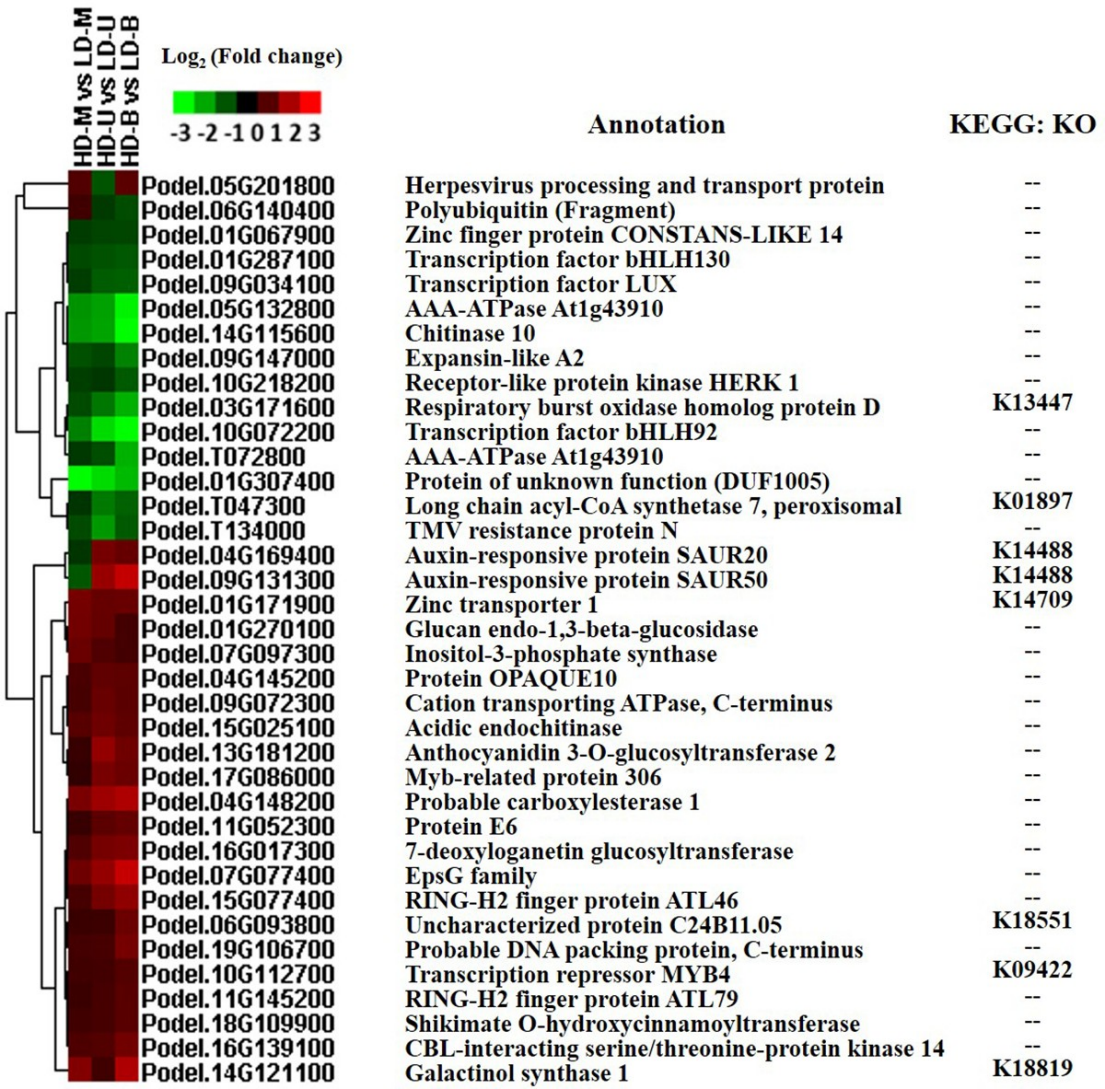
Heat map of the expression levels of the 37 planting density-regulated differentially expressed genes (DEGs). DEGs were identified by comparing samples from poplar trees planted under high (HD) and low (LD) density. The samples were taken from three vertical positions for comparisons: upper (U), middle (M), and bottom (B).

Based on the Venn diagram results, it was expected that the density stress would affect the expression levels of genes. There were 37 DEGs found at all three positions when comparing HD and LD. Among these, a total of 33 DEGs that displayed similar expression patterns were screened out, of which 20 were upregulated and 13 were downregulated under HD (Fig 6). These DEGs included the basic helix-loop-helix (*bHLH*) transcription factors (TFs), LUX ARRHYTHMO (*LUX*), zinc finger protein, and RING-H2 finger protein. In addition, there were four genes that displayed inconsistent expression trends: two auxin-responsive proteins (small auxin-up RNAs–SAURs), one herpesvirus processing and transport protein, and one polyubiquitin. Surprisingly, most of these genes were not annotated by KEGG, and so there was limited information on the metabolic pathways that could be involved.

### DEGs related to light response and photosynthesis

**Fig 7:**
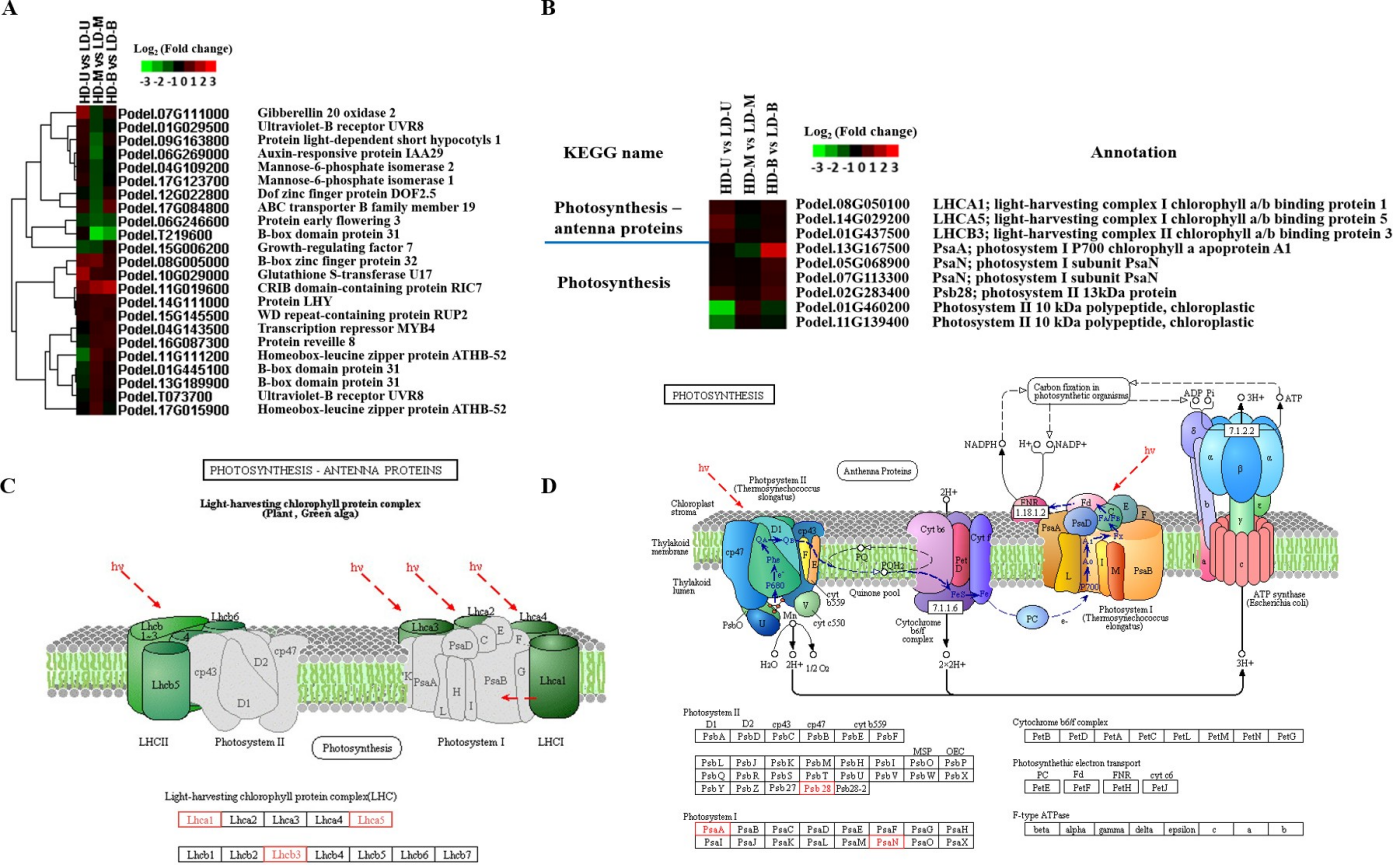
Expression of DEGs involved in response to light and photosynthesis. Differentially expressed genes (DEGs) were identified by comparing samples from poplar trees planted under high (HD) and low (LD) density. The samples were taken from three vertical positions for the comparisons: upper (U), middle (M), and bottom (B). (A) A heat map of the expression levels of DEGs that were found to be involved in response to light stimuli. (B) A heat map of the expression levels of DEGs involved in photosynthesis. (C) The structure of photosynthesis-antenna proteins. (D) The structure and mechanism of photosynthesis.

When comparing HD and LD at the middle position, 23 DEGs were found in the “response to light stimulus” GO subcategory. For nine-year-old poplar grown under HD, trees are already in a canopy closure state. The light conditions through the whole canopy space were obviously weakened, especially at the middle and bottom positions of the trees. The expression levels of the 23 light stimulus-related DEGs displayed two opposing trends at the middle position. Under HD, 11 of the genes were downregulated and 12 upregulated (Fig 7A). In contrast, most of the DEGs found at the upper and bottom positions were upregulated under HD.

From the KEGG enrichment results, nine photosynthesis-related genes were observed, including three “photosynthesis-antenna proteins” and six “photosynthesis genes” (Fig 7B). The expression of the three photosynthesis-antenna proteins (LHCs) were mostly upregulated under HD; it was only at the middle position that no significant differential expression was observed. Meanwhile, all of the photosystem-related genes were also upregulated under HD, except for two photosystem II 10 kDa polypeptides. The nine photosynthesis-related genes were cast into either the “photosynthesis-antenna proteins” and “photosynthesis” KEGG pathways (Fig 7C and 7D).

### DEGs related to the auxin/indole-3-acetic acid (AUX/IAA) signaling response

**Fig 8:**
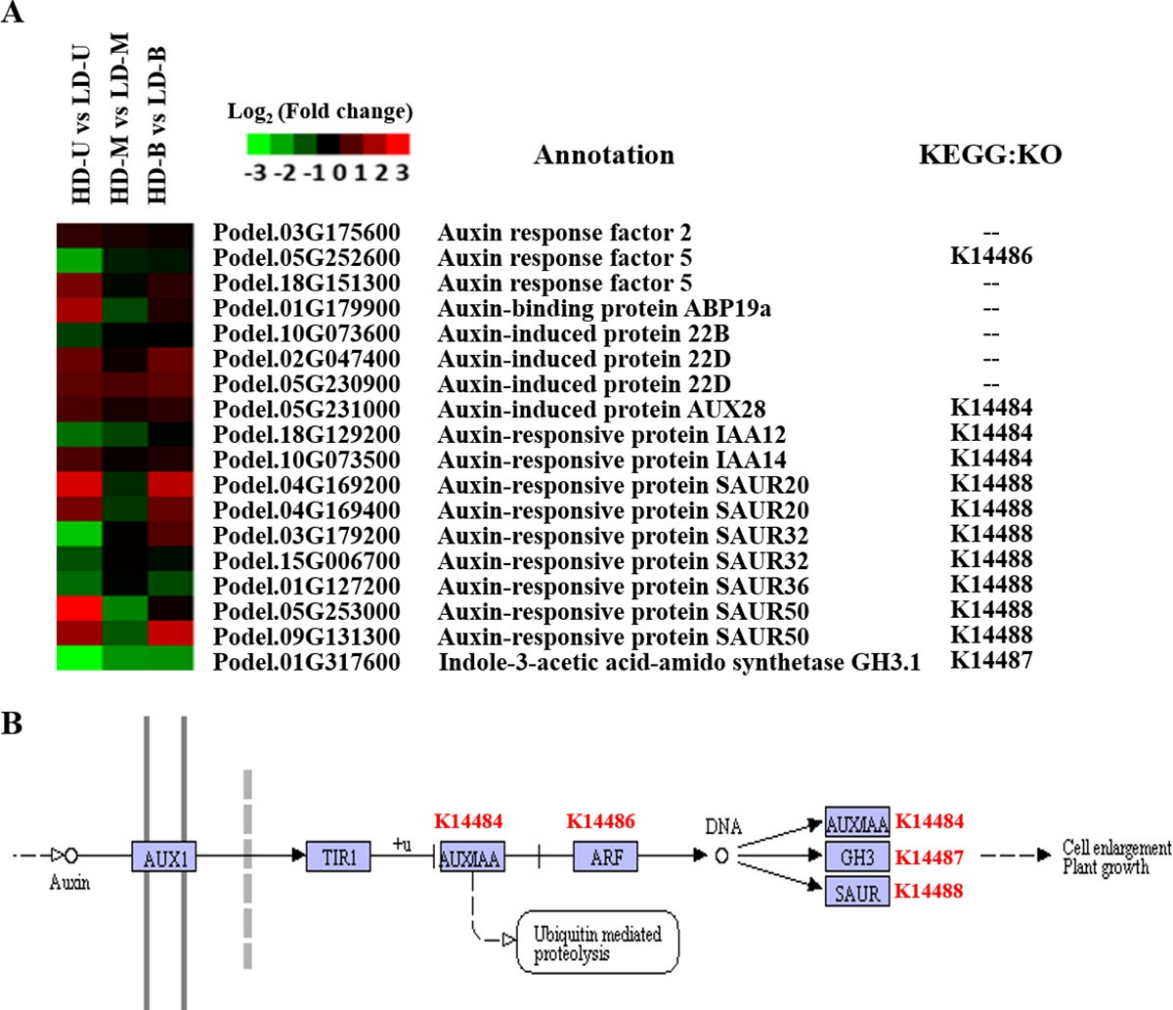
DEGs related to the auxin/indole-3-acetic acid (AUX/IAA) signal transduction pathway. Differentially expressed genes (DEGs) were identified by comparing samples from poplar trees planted under high (HD) and low (LD) density. The samples were taken from three vertical positions for the comparisons: upper (U), middle (M), and bottom (B). (A) A heat map of expression levels of the DEGs related to AUX/IAA. (B) The DEGs involved in the AUX/IAA signal transduction pathway.

At the upper position, 18 AUX/IAA-related genes were differentially expressed between HD and LD. Of these, seven genes were significantly downregulated under HD (Fig 8A). Using the KEGG annotation results, it was found that 18 AUX/IAA-related genes were mainly involved in AUX/IAA signal transduction (Fig 8B). No genes were found to be associated with AUX/IAA biosynthesis. A similar overall trend was observed at the bottom position. In contrast, the middle position showed different expression trends; the AUX/IAA-related genes were downregulated under HD when compared to LD. Additionally, one Gretchen Hagen (*GH3*.*1*) gene was significantly downregulated under HD in all three comparisons. Thus, it could be suggested that different planting densities had a significant effect on the AUX/IAA signal transduction pathway, and the response at each vertical position was also distinctive.

### DEGs involved in carbon and nitrogen metabolism

**Fig 9:**
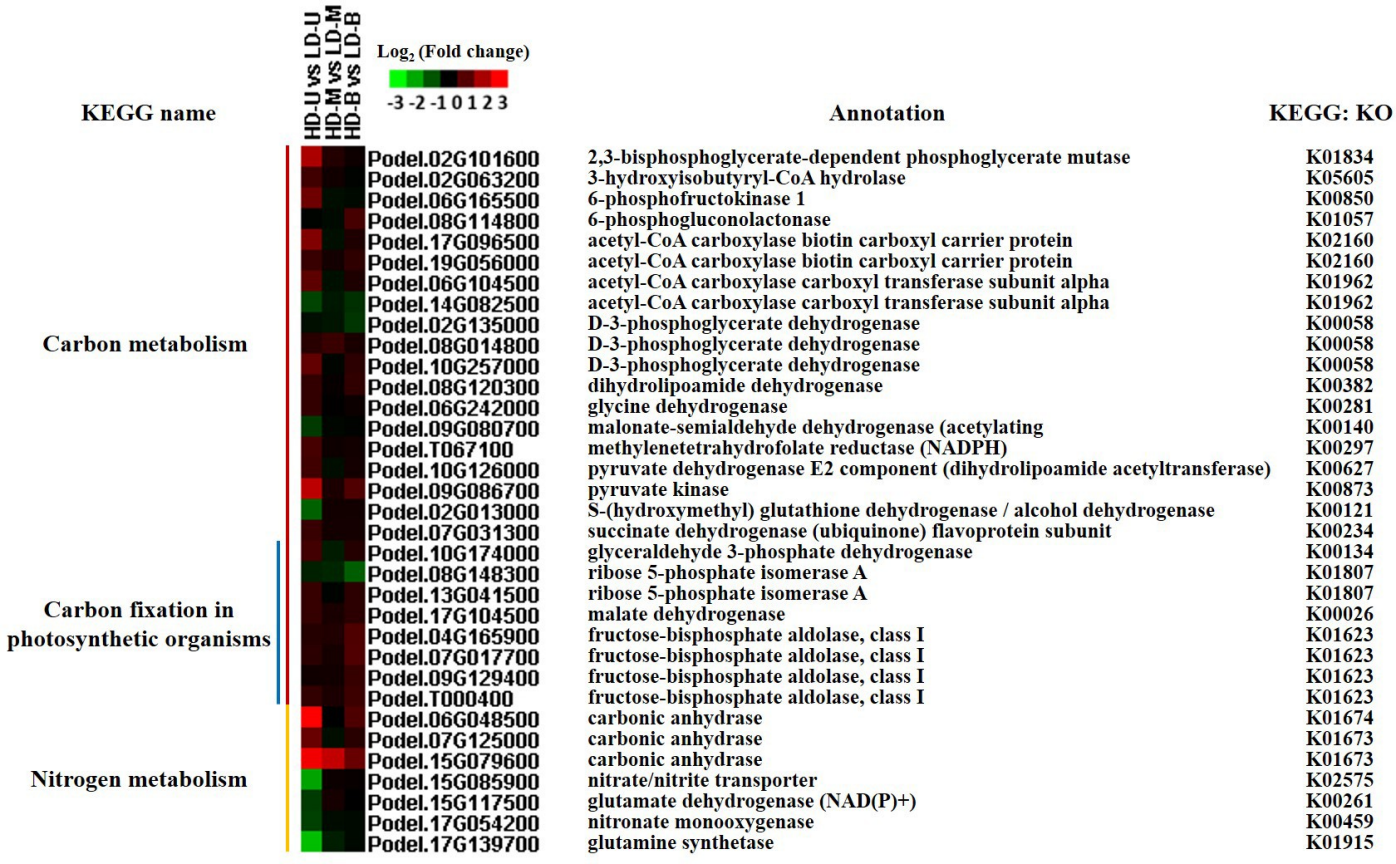
Expression of DEGs involved in carbon and nitrogen metabolism. Differentially expressed genes (DEGs) were identified by comparing samples from poplar trees planted under high (HD) and low (LD) density. The samples were taken from three vertical positions for the comparisons: upper (U), middle (M), and bottom (B).

**Fig 10:**
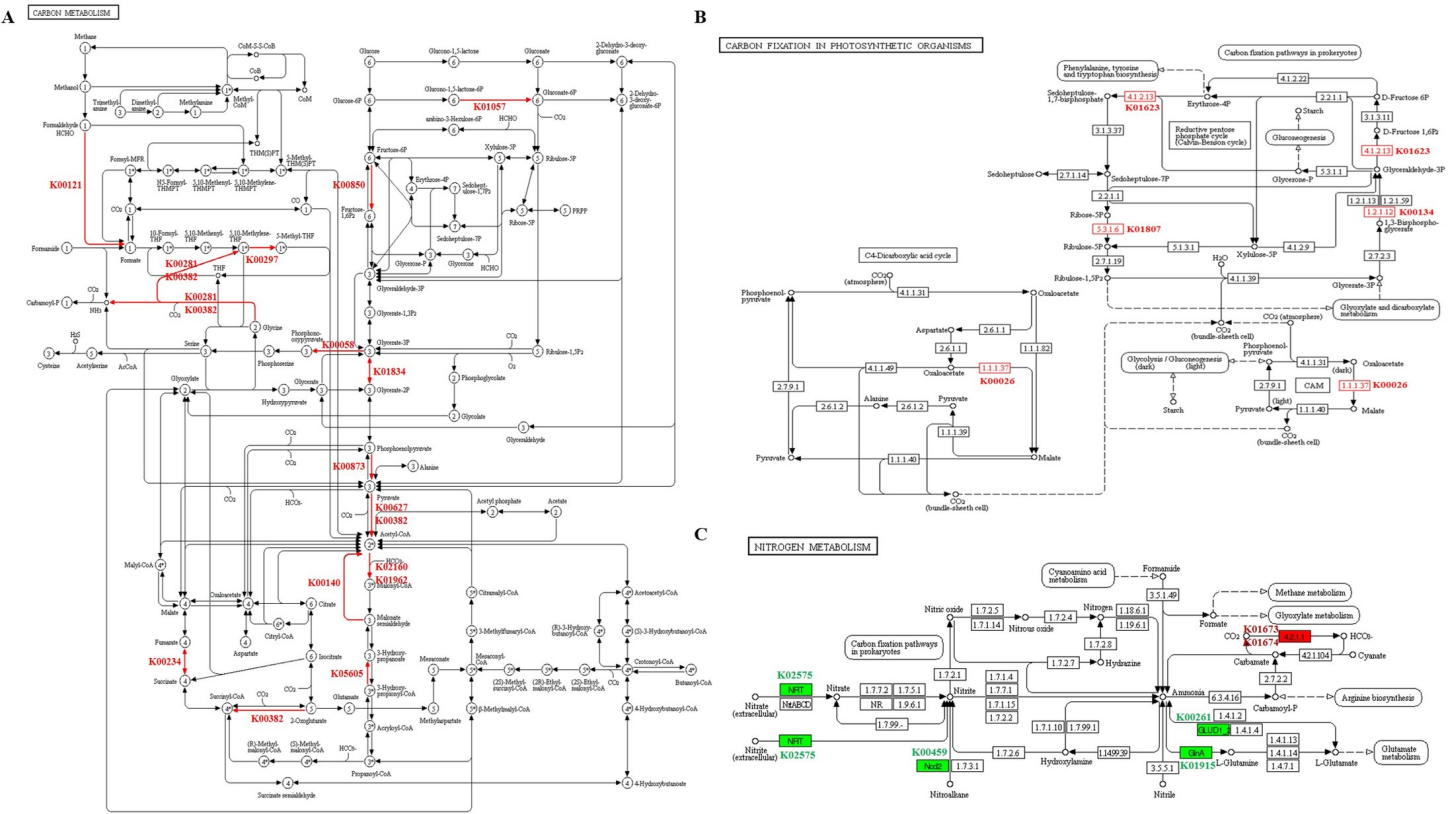
The role of identified DEGs in carbon and nitrogen metabolism pathways. (A) DEGs involved in the “carbon metabolism” pathway. (B) DEGs involved in the “carbon fixation in photosynthetic organisms” pathway. (C) DEGs involved in the “nitrogen metabolism” pathway.

Carbon and nitrogen metabolism are critical to plant growth and development. Some DEGs that participated in carbon and nitrogen metabolism were identified from transcriptome profiles, including 19 “carbon metabolism” genes, 8 “carbon fixation in photosynthetic organisms” genes, and 7 “nitrogen metabolism” genes (Fig 9). Furthermore, detailed information on the corresponding metabolic pathways in which these genes are involved is shown in Fig 10. When comparing HD to LD at all three positions, the overall trend in expression of these genes was upregulation under HD. Four nitrogen metabolism genes (Podel.15G085900, Podel.15G117500, Podel.17G054200 and Podel.17G139700) were downregulated under HD at the upper position. The genes displaying an upregulation trend, showed large changes in different density comparisons, which suggests that the different planting densities influenced carbon and nitrogen metabolism.

### Validation of the RNA-Seq data by qRT-PCR

**Fig 11:**
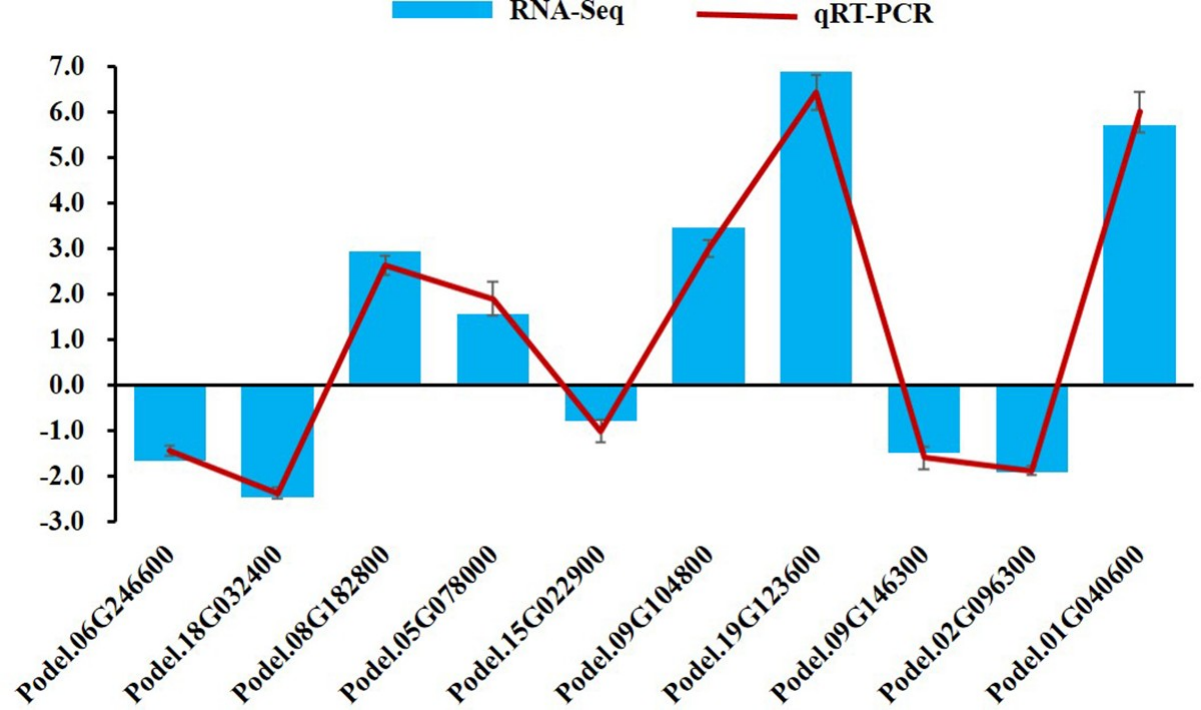
Comparison of expression patterns of 10 randomly selected genes based on the results of RNA-seq and qRT-PCR. The x-axis represents the 10 randomly selected genes and the y-axis represents the value of log2 (relative expression). The blue histogram represents results from RNA-Seq and the red line represents results from qRT-PCR. Error bars indicate the standard deviation of the qRT-PCR data.

To further verify the accuracy of the RNA-Seq data, we conducted qRT-PCR assays using the same samples as those used for RNA-seq. Ten genes were randomly selected from the planting density comparison at the middle position, for expression level analysis using qRT-PCR. The findings displayed good correlation with the results of the RNA-seq (Fig 11), confirming the reliability of the RNA-Seq data.

## Discussion

The ability of trees to maintain a high level of productivity under an adequate planting density is a determinant factor for increasing timber yield. With increasing establishment of poplar plantations, further research on how trees adapt to their growth environment at the molecular level is necessary to secure optimum timber yield and quality. For the nine-year-old poplar, trees grown under HD are already in a canopy closure state; the DBH was found to be highly significantly different between the two planting densities. Here, the whole-genome RNA-Seq was utilized to profile transcript levels in mature poplar trees planted under HD and LD. The results revealed that a large amount of DEGs (4,004) existed at three positions when comparing HD with LD. In addition, it was found that the vertical position had a significant effect on gene expression when comparing planting densities. Guo et al. sought out 205 genes that were considered to be differentially expressed in *Arabidopsis* grown under different planting densities [7]. Another study found that 221 genes exhibited differential expression in response to density stress in barley [28]. These results suggest that transcriptome profiles are influenced by changes in planting density in both annual herbaceous and perennial woody plants.

### Expression of DEGs related to transcription factors

An adequate planting density is an important regulator for plant growth and development. In the present study, 33 of 37 density-regulated DEGs showing similar expression profiles were identified from all three positions when HD was compared with LD. These included RING-H2 finger protein and TFs (*bHLH*, *LUX* and *MYB*). Ring finger proteins play a pivotal role in regulating growth and developmental processes, hormone signaling pathways and defense responses against biotic and abiotic stresses in plants [29]. The *bHLHs* play important roles in regulating plant growth and development, and participate in abiotic-stress responses [30]. The expression of *bHLH92* has been shown to be strongly induced by salt, drought, osmosis, cold and other stresses. Meanwhile, transgenic *Arabidopsis* plants overexpressing *bHLH92* exhibited a strong salt stress tolerance [31]. Li et al. isolated the *OrbHLH001* gene from rice and found that *Arabidopsis* plants overexpressing *OrbHLH001* displayed an improved resistance to salt and freezing [32]. In our study, two *bHLH* genes (*bHLH92* and *130*) were observed to be downregulated under HD compared with LD. MYB proteins play an important role in plant signal transduction and response to biotic and abiotic stresses. The *R2R3-MYB* gene has been shown to be involved in plant responses to environmental stresses such as drought, salt and cold [33,34]. Given the important roles of these TFs in other plants, we hypothesized that these DEGs might be affected by planting density, particularly the MYB-related proteins that were upregulated under HD in all three comparisons. Meanwhile, these genes that were regulated by planting density stress, have been found to be involved in the pertinent environmental response in other studies.

### Expression of DEGs related to photosynthesis

Little is known about dynamic gene expression in trees in response to light under different planting densities. Therefore, understanding these changes on a molecular basis is critical. From our transcriptomic data, 23 DEGs were found to respond to light stimuli, three “photosynthesis-antenna proteins” and six “photosynthesis” genes were identified, of which most were upregulated under HD. In the present study, the light responsive Ultraviolet (UV)-resistance locus 8 (*UVR8*) and *MYB4* genes were upregulated in HD compared with LD. *UVR8*, a light receptor, can detect specific ultraviolet wavelengths [35]. Moreover, MYBs have been found to regulate stomatal number and size during photosynthesis, and are known to participate in photosynthesis in response to light stress [36–38]. Light-harvesting complex I/II chlorophyll a/b binding proteins (LHCs), known as light-harvesting antennae, are essential for photosynthesis in plants, and form the basis of photosynthesis in eukaryotes [39]. LHCs are a membrane protein in plant photosystem I that binds to chlorophyll and forms light-harvesting chlorophyll protein complexes, and mainly involved in the capture and transfer of light energy in photosynthesis [40]. Photosystems I and II are two large protein cofactor complexes situated in the thylakoid membrane [41]. Photosystem I, serves as a sunlight energy converter, catalyzing one of the initial steps in driving oxygenic photosynthesis [42]. Photosystem II is the site of oxygenic photosynthesis and performs a series of light-induced electron transfer reactions [43]. St. Pierre et al. found that transcripts encoding the photosystem II 10 kDa protein in barley and maize were all upregulated under HD compared with LD [28]. High planting density conditions have a similar effect on growth to shading in the field [44]. That is, plants grown under HD display shade avoidance. Shade avoidance syndrome (SAS)-related genes, such as phytochrome B (*PHYB*), non-phototropic hypocotyl (*NPH*), class II homeodomain-leucine zipper (*HD-ZIP* II) and parotid isoelectric focusing (*PIF*) protein genes, have been found to be involved in the shade avoidance response [45–47]. However, in this study, we failed to detect all the probes for these genes, perhaps because we did not utilize the extreme treatments of either complete darkness or low red to far red light ratio (R:Fr). Although the ratio of R:Fr should be higher under LD, there is still far red light for the plants to detect in the surrounding environment. Considering that most of the genes from our transcriptomic data were upregulated under HD, photosynthesis may be activated. This suggests that trees may have a self-regulating mechanism to maintain normal growth and development under higher planting densities.

### Expression of DEGs related to auxin signaling

Phytohormones have long been considered as essential endogenous molecules participating in regulation of plant growth and development, and tolerance to diverse stresses [48]. Hormonal pathways are often relevant to density stress [28]. It has been demonstrated that auxins signaling pathways are mediated by phytochrome and inhibit lateral shoot growth in plants [49,50]. From the KEGG enrichment analyses, genes involved in the “plant hormone signal transduction” pathway appeared at two positions (upper and bottom) when comparing HD and LD (Fig 5), which indicates that the planting density significantly influenced hormonal gene expression. Eighteen AUX/IAA-related genes that were differentially expressed between HD and LD were screened out from the transcriptomic results. These auxin-related genes were mainly auxin response factors (ARFs), auxin-binding proteins (ABPs), auxin-induced proteins, auxin-responsive proteins and IAA-amido synthetases. Overall, most of these genes were upregulated under HD when compared to LD at the upper and bottom positions, while the expression at the middle position was downregulated for consistency. Inconsistencies can be observed in the expression trends of these genes between HD and LD at different positions.

ARFs regulate the expression of auxin response genes by binding to auxin response elements in the promoter regions of the auxin response genes [51]. In the plant density comparison at the upper position, two ARFs (Podel.03G175600 and Podel.18G151300) were upregulated, and one (Podel.05G252600) was downregulated under HD. SAURs have been mainly found to be expressed in the elongating tissues and to function in auxin-mediated cell elongation [52]. For our data, seven SAURs showed two different expression trends.; however, most of the SAURs were upregulated under HD. One *GH3*.*1* gene (Podel.01G317600) was significantly downregulated under HD at all three positions. Previous studies have revealed that the GH3 proteins modulate multiple biological processes, including photomorphogenesis [53], light signaling, auxin signaling and auxin homeostasis in *Arabidopsis* [54,55]. The rice Leaf Inclination 1 (*LC1*) gene encodes the IAA-amido synthetase *OsGH3-1*, which catalyzes the combination of excessive IAAs with multiple amino acids to maintain auxin homeostasis, and regulates leaf angle by altering the cell elongation [56]. Under high planting density, the leaf angle of plants is bound to change to a certain extent. Therefore, we speculate that the *GH3*.*1* gene (Podel.01G317600) may be involved in this process. Based on these findings, we conjecture that the different expression trends between HD and LD at the three positions may be to balance the level of auxin, and improve the growth and development of the plant when growing under high planting density.

### Activation of carbon and nitrogen metabolism

Carbon and nitrogen metabolism, the basic metabolic pathways in plants, not only affect plant growth and development, but also determine the yield [57]. Under different planting densities, the microhabitats in which plants are grown exhibit differences in light, temperature, water, gases, and nutrients. It has been found that environmental factors (light, temperature and nutrient status) could influence the carbon and nitrogen metabolism process, and alter the growth rhythms of plants [58–60]. From our transcriptomic results, a number of DEGs involved in carbon and nitrogen metabolism were identified; overall these genes showed an upregulation trend under HD compared with LD at the three positions.

Carbonic anhydrase (CA) can accelerate the diffusion of inorganic carbon to the active site of carboxylase and increases the fixation rate of CO_2_ [61]. Salicylic acid binding protein 3 (SABP3), a chloroplastic CA, has been shown to display antioxidant activity and hypersensitivity to defense response in tobacco [62]. In our study, three CA genes were strongly upregulated under HD, indicating that the CA genes responded to high density stress. Nitrate, as the main inorganic nitrogen source for plant growth and development, is not only a nutrient for plants, but can also act as a signaling molecule to regulate plant morphogenesis, physiological responses and gene expression [63–65]. Guo et al. showed that nitrate availability is the main factor limiting agricultural productivity under high density conditions [7]. Bouguyon et al. found that the nitrate/nitrite transporter (*NRT1*.*1*) gene was not only involved in nitrate response and transport, but also in plant response to stress [65]. Glutamine synthetase (GS) is a key enzyme in plant nitrogen assimilation, catalyzing the conversion of ammonium to glutamine [66], and plays an important role in plant growth and yield formation [67]. We found that two genes related to nitrogen metabolism (*NRT*-Podel.15G085900 and *GS*-Podel.17G139700), were significantly downregulated under HD at the upper position. However, no differences in expression of the two genes were observed for comparisons at the middle and bottom positions. Chen et al. analyzed the effects of plant population on the dynamic changes of carbon and nitrogen content, found that high plant density had a significant effect on carbon metabolism [68]. Based on our results, we concluded that changes in the expression of genes related to carbon and nitrogen metabolism, may enable the plants to adapt to high planting density.

According to our results, carbon and nitrogen metabolism genes were mostly upregulated under HD. We propose that normal growth of poplar trees could proceed under planting density stress via the activation of carbon and nitrogen metabolisms.

## Conclusions

The whole-genome RNA-seq of leaves from perennial woody poplar provided insight into gene expression patterns under high and low planting densities. Overall, 4,004 DEGs emerged in the comparison of HD with LD, with 37 density-related DEGs found at all three positions. Moreover, several light-related genes, which were mostly upregulated under HD, were also observed. These genes may therefore play an important role in the response to light under different positions and planting densities. A series of AUX/IAA-related genes that showed diverse expression trends were also analyzed. Meanwhile, genes involved in carbon and nitrogen metabolism were identified. Of these genes, those displaying increased expression under HD, may be indicative of the plants’ ability to adapt to density stress. These findings could increase our molecular understanding of the effect of planting density on gene expression and provide a reference for future research on planting density-regulated genes. In the future, we hope to achieve optimal expression of the most important functioning genes by regulating planting density. This should enable us to obtain the maximum yield under high density.

## Supporting information

S1 Fig Heatmap showing the correlation coefficients between different samples. The correlation coefficients were calculated using log2 (FPKM). The color represents the correlation coefficient values (the redder the color, the higher the correlation, the less red the color, the lower the correlation). LD: low planting density. HD: high planting density. U: upper, M: middle, and B: bottom vertical sampling positions.
(TIF)

S1 Table Primers used for qRT-PCR analysis of genes selected from the RNA sequencing data. (XLSX)

S2 Table Summary of the RNA sequencing data. (XLSX)

S3 Table Summary of the mapping results of RNA sequencing data. (XLSX)

S4 Table Distribution of mapped reads over different genomic regions. (XLSX)

S5 Table Statistical distribution of FPKM interval values in each sample. (XLSX)
