# Supplementary figures and images for: Transcriptome profiling revealed diverse gene expression patterns in poplar (Populus × euramericana) under different planting densities
Source: PLoS One. 2019 May 29;14(5):e0217066. doi: 10.1371/journal.pone.0217066 (PMC6541269; doi:10.1371/journal.pone.0217066)

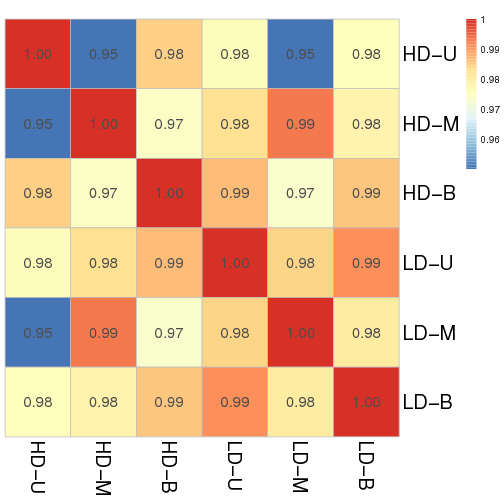

Supplement: S1 Fig — The correlation coefficients were calculated using log2 (FPKM). The color represents the correlation coefficient values (the redder the color, the higher the correlation, the less red the color, the lower the correlation). LD: low planting density. HD: high planting density. U: upper, M: middle, and B: bottom vertical sampling positions. (TIF) [file pone.0217066.s001.tif]
